# Supplementary material for: Development and Characterization of a Cancer Cachexia Rat Model Transplanted with Cells of the Rat Lung Adenocarcinoma Cell Line Sato Lung Cancer (SLC)
Source: Biomedicines. 2023 Oct 18;11(10):2824. doi: 10.3390/biomedicines11102824 (PMC10604092; doi:10.3390/biomedicines11102824)
Supplement: Supplementary file 1 [file biomedicines-11-02824-s001.zip › biomedicines-2666424-supplementary.pdf]

Supplementary Materials

Development and Characterization of a Cancer Cachexia Rat Model Transplanted with Cells of the Rat Lung Adenocarcinoma Cell Line Sato Lung Cancer (SLC)

Eiji Kasumi *, Miku Chiba, Yoshie Kuzumaki, Hiroyuki Kuzuoka, Norifumi Sato and Banyu Takahashi

R&D Laboratories, EN Otsuka Pharmaceutical Co., Ltd., Hanamaki 025-0312, Japan; sato.norifumi.a@otsuka.jp (N.S.)

* Correspondence: kasumi.eiji@otsuka.jp; Tel.: +81-198-30-1883; Fax: +81-198-30-1819

Table S1. mRNA-specific primer pairs for RT-PCR.

Rat mRNA (NCBI ID)	Sequence (5'-3')	
MuRF1	Forward	GGAGAAGCTGGACTTCATCG
(AY059627.1)	Reverse	CTTGGCACTCAAGAGGAAGG
atrogin-1	Forward	GAACATCATGCAGAGGCTGA
(AY059628.1)	Reverse	GTAGCCGGTCTTCACTGAGC
GAPDH	Forward	CCCCCAATGTATCCGTTGTG
(AB017801.1)	Reverse	TAGCCCAGGATGCCCTTTAGT

RT-PCR, reverse transcriptase polymerase chain reaction; MuRF1, muscle-specific ring finger protein 1; GAPDH, glyceraldehyde-3-phosphate dehydrogenase.

Table S2. Nutritional composition of the liquid diet used in the tube feeding experiment.

Components	Calories (/100 kcal)
Proteins (g)	3.5
Fats (g)	3.5
Carbohydrates (g)	13.7
Minerals	
Sodium (mg)	80.0
Potassium (mg)	150.0
Calcium (mg)	53.3
Magnesium (mg)	20.0
Phosphorus (mg)	53.3
Chloride (mg)	136.0
Iron (mg)	0.9
Zinc (mg)	1.5
Manganese (mg)	0.2
Copper (mg)	0.1
Selenium (μg)	1.6
Chromium (μg)	0.9
Molybdenum (μg)	1.9
Vitamins	
Vitamin A (μg RE [1])	75.0
Vitamin D (μg)	0.5
Vitamin E (mg)	3.0
Vitamin K (μg)	7.0
Vitamin B1 (mg)	0.2
Vitamin B2 (mg)	0.2
Vitamin B6 (mg)	0.2
Vitamin B12 (μg)	0.6
Vitamin C (mg)	15.2
Nicotinamide (mg)	2.0
Pantothenic acid (mg)	0.5
Folic acid (μg)	20.0
Biotin (μg)	15.2
Choline (g)	0.1

[1] RE, retinol equivalents.
